# Ethiopian Population Dermatoglyphic Study Reveals Linguistic Stratification of Diversity

**DOI:** 10.1371/journal.pone.0126897

**Published:** 2015-06-04

**Authors:** Seile Yohannes, Endashaw Bekele

**Affiliations:** 1 Department of Biology, Jigjiga University, Jijiga, Ethiopia; 2 Department of Microbial, Cellular, & Molecular Biology, Addis Ababa University & Centre for Human Genetic Diversity, Addis Ababa, Ethiopia; National Cheng-Kung University, TAIWAN

## Abstract

The manifestation of ethnic, blood type, & gender-wise population variations regarding Dermatoglyphic manifestations are of interest to assess intra-group diversity and differentiation. The present study reports on the analysis of qualitaive and quantitative finger Dermatoglyphic traits of 382 individuals cross-sectionally sampled from an administrative region of Ethiopia, consisting of five ethnic cohorts from the Afro-Asiatic & Nilo-Saharan affiliations. These Dermatoglyphic parameters were then applied in the assessment of diversity & differentiation, including Heterozygosity, Fixation, Panmixia, Wahlund’s variance, Nei’s measure of genetic diversity, and thumb & finger pattern genotypes, which were inturn used in homology inferences as summarized by a Neighbour-Joining tree constructed from Nei’s standard genetic distance. Results revealed significant correlation between Dermatoglyphics & population parameters that were further found to be in concordance with the historical accounts of the ethnic groups. Such inductions as the ancient north-eastern presence and subsequent admixure events of the Oromos (PII= 15.01), the high diversity of the Amharas (H= 0.1978, F= 0.6453, and P= 0.4144), and the Nilo-Saharan origin of the Berta group (PII= 10.66) are evidences to this. The study has further tested the possibility of applying Dermatoglyphics in population genetic & anthropologic research, highlighting on the prospect of developing a method to trace back population origins & ancient movement patterns. Additionally, linguistic clustering was deemed significant for the Ethiopian population, coinciding with recent genome wide studies that have ascertained that linguistic clustering as to being more crucial than the geographical patterning in the Ethiopian context. Finally, Dermatoglyphic markers have been proven to be endowed with a strong potential as non-invasive preliminary tools applicable prior to genetic studies to analyze ethnically sub-divided populations and also to reveal the stratification mechanism in play.

## Introduction

The structural arrangements & patterns of the epidermal ridges on the finger tips (fingerprints) are under the control of genetic as well as prenatal environmental factors [1], and broadly studied under a field of science known as Dermatoglyphics. They are of special interest to forensic experts, anthropologists, geneticists, and physicians alike [2, 3]. The study of Rife [4] served as a stepping stone from an anthropological point of view, following which, several researchers from nations worldwide have expounded Dermatoglyphic profiles for most of their representative ethnic groups, employing them to infer important population genetic parameters [5, 6, 7].

Ethiopia is a very diverse country, both with respect to its ethnic profile as well as its recognized linguistic diversity, with about 83 different languages & over around 200 different dialects represented [8]. Its linguistic diversity can easily be observed by the presence of three branches of the Afro-Asiatic language family (Semitic, Cushitic, Omotic), as well as Nilo-Saharan representatives, not to mention the variety of languages within each of these classes. Genetic studies have shown that the diversity of the Ethiopian population shows linguistic clustering, to an extent that such linguistic clustering being more important than the geographical structure for this population [9].

Despite this, Dermatoglyphic studies in Ethiopia are very scarce, with only one citable published literature available [10]. The current study has assessed the Dermatoglyphic patterns of five of the main ethnic groups found in one of the nine administrative regional states, and has further used the generated data in diversity & homology inferences for the different groups.

## Materials and Methods

### Ethical Clearance

The study protocol was reviewed & approved by the Ethical Review Committee of Department of Microbial, Cellular and Molecular Biology of the Addis Ababa University. Permission was also granted by the governmental administrative office of the study area and Federal Police Commission of Benishangul Gumuz Regional State.

### Study Area and Subjects

The study was carried out in the Benishangul-Gumuz Regional state of Ethiopia, located in the mid-western part of the country. Sampling took place in the town of Assosa, located between 10°04N latitude and 34°31E longitude, with an elevation of 1570 meters. The study was performed as a cross-sectional study in which initially 450 individuals were systematically sampled from the town of Assosa, following which, ethnic groups with workable sample sizes were retained, while those with very low sample sizes, those with mixed ethnicities (different maternal & paternal lines), plus those with illegible/incomplete Dermatoglyphic sheets were left out of the analysis. Thus, the total retained sample population was 382 individuals, which included representatives from both major linguistic families spoken in Ethiopia, vis-à-vis the Afro-Asiatic Family & the Nilo-Saharan Family. The ethnic representatives from the three branches of the Ethiopian Afro-Asiatic Language family were the Semitic (Amhara, Tigray), the Cushitic (Oromo), and the Omotic (Shinasha), while those from the Nilo-Saharan Family were represented by the Berta group. The study participants were explained of the purpose, relevance, procedures, & technical details of the study prior to involvement. Willing subjects then verified compliance by signing a formal consent form.

### Dermatoglyphic Variables

**Fig 1:**
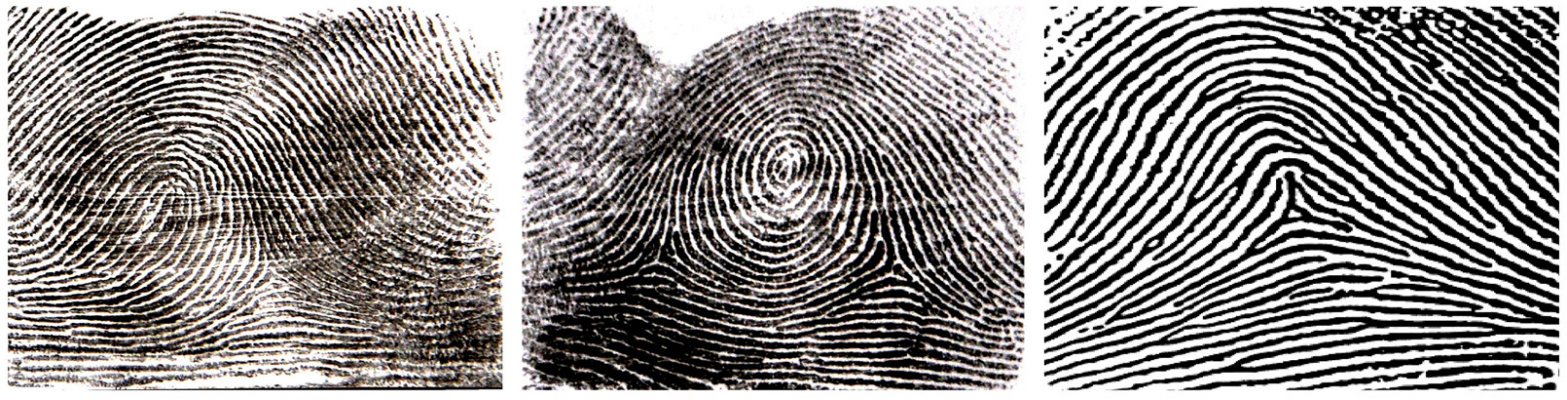
The three basic fingerprint patterns: from left to right- Loop, Whorl, and Arch fingerprint pattern types. Ridge counts are determined by drawing lines that connect the core/centre of the patterns with the respective delta (triradius), thus yielding two ridge counts for the whorls, one for the loops, and none for the arches (since they lack a delta).

Fingerprints were obtained by using the ink-and-paper method [11], and subjects were blood-typed using ABO & Rh (D/d) monoclonal antibodies, performed by a trained professional clinical nurse. Dermatoglyphic traits were defined & quantified according to the standard methods [11, 12]. The quantitative Dermatoglyphic variables included in this study include the total finger ridge count (TFRC- sum of the higher ridge counts of the 10 fingers), the Absolute Ridge Count (ARC- sum of all possible ridge counts of the 10 fingers), and the Radial Ridge Count (RRC- sum of the ridge counts with a direction of opening towards the radial bone /thumb side/ of the 10 fingers). Similarly, the qualitative variables include the pattern types, classified as Loops (with one delta & one core), Whorls (with one/two cores and two deltas), and Arches (with no true core or delta), as depicted in Fig 1. For the ridge counting process, following manual counting using a magnifying lens by the researchers, the Semi-Automated *Ridge Counter* Software [13] was further employed to improve accuracy.

Three pattern indices [14, 15] were calculated: the Pattern Intensity Index (PII- the pattern intensity of each finger is defined as the number of Triradii present in the pattern: Loops & Tented Arches have one; Plain, Double Loop, & Central Pocket Whorls have two; Accidentals have up to three. Thus the pattern intensity index (PII) of an individual is the sum total of the pattern intensities of each of the fingers), the Dankmeijer’s Index (DI- the proportion of Arches relative to the Whorls in the overall population/grouping, calculated as 100 times arches divided by the whorls), and the Furuhata’s Index (FI- the proportion of Whorls relative to the proportion of Loops in the overall population/grouping calculated as 100 times loops divided by the whorls). In addition, the Thumb & Finger genotypes (Table A in S1 File) were estimated as per the proposed inheritance model [16].

### Statistical Analysis

Chi-Square tests, one-way ANOVA, and Post-Hoc Comparisons as per the standards [17,18], all with significances at the 5% level, were employed for statistical analysis using the SPSS (version 20) package.

### Diversity

Diversity analysis was done using Heterozygosity, Fixation /Panmixia/, Wahlund’s Variance [19, 20], and Nei’s measure of genetic diversity [21]. Further, Nei’s standard (D_A_) distance [22] was calculated on eight variables: the seven allelic frequencies from polymorphic loci related to thumb & finger pattern genotypes and the fingerprint pattern frequencies (Table B in S1 File), which was used to draw a Neighbour-Joining phylogenetic tree applying the *POPTREE* 2 software [23], and tree topology was edited for convenience using the *MEGA version 4* software [24].

## Results and Discussions

### Dermatoglyphic Variables

The distributions in the various groupings of fingerprint patterns, mean ridge counts and other relevant data are summarized herewith (see Tables 1, 2, 3, & 4). In the overall sampled population, the most common pattern type scored were the Ulnar Loops (54.7%), followed by the Whorls (37%), while the Arches have a relatively lower frequency (7.7%). The least common patterns observed were the Radial Loops (< 1%). The mean PII value was 12.94, a value which, according to Rife [4], is within the generally expected range for the area in and around Ethiopia. Similarly, the mean DI and FI values were 20.71 & 67.00 respectively.

**Table 1:** TRC distribution pattern, showing mean, standard deviation, and skew of the normal distribution.

Variable	Mean	S.D.^a^	Skewness	
			Statistics (Value)	S.E.^b^
TFRC	129.55	42.01	-0.55	0.22

The TFRC (mean & standard deviation) of the sample, showing the normal distribution of this quantitative variable, with a slight negative skew.

^a^ Standard Deviation.

^b^ Standard Error.

**Table 2:** Fingerprint pattern and ridge count distributions among the ethnic groups.

		Distribution (%)			Ridge Counts (Mean)		
Group	N^a^	A^b^	L^c^	W^d^	TFRC	AFRC	RRC
**Overall**	382	7.67	55.29	37.04	129.55	175.68	43.55
**Amhara**	91	6.7	65.82	27.47	123.91	149.35	27.70
**Berta**	68	18.68	56.03	25.29	90.04	101.16	13.56
**Oromo**	104	4.23	41.44	54.33	157.42	235.58	81.61
**Shinasha**	62	4.03	52.42	43.55	146.71	188.38	46.71
**Tigray**	57	6.32	65.96	27.72	125.08	146.46	24.21
**Statistic**		χ2 (d.f)^e^ = 355.86 (8)			F = 14.31	F = 20.61	F = 21.37
**p** ^**^f^**^		**<0.0000001**			**<0.00001**		

The Nilo-Saharan Berta group manifested significantly higher Arches. The two Semitic groups manifested comparable frequencies for all three pattern types, as have the Cushitic & Omotic representatives. Loops were found in highest percentages among the Semitics, while the least frequencies were observed for the Oromos. Whorls on the other hand were recorded in the highest frequencies for the Oromos. Regarding the TFRC, ARC, & RRC distributions, the Oromos manifested the highest values, while the Bertas recorded the lowest values, coinciding with the high whorl & arch patterns respectively in the two groups.

^a^ Sample Size.

^b^ Arch.

^c^ Loop.

^d^ Whorl.

^e^ Degrees of Freedom.

^f^ Significance.

**Table 3:** Fingerprint pattern indices distributions among the ethnic groups.

	Pattern Indices		
Group	PII^a^	DI^b^	FI^c^
**Overall**	12.94	20.71	67.00
**Amhara**	12.08	24.40	41.74
**Berta**	10.66	73.84	45.14
**Oromo**	15.01	7.79	131.09
**Shinasha**	13.95	9.26	83.08
**Tigray**	12.14	22.78	42.02

The distribution of indices related to fingerprint patterns, showing that the highest & lowest pattern intensities (PII) were scored by the Cushitic Oromos and the Nilo-Saharan Bertas. Similarly, the two Semitic groups manifested comparable indices.

^a^ Pattern Intensity Index.

^b^ Dankmeijer’s Index.

^c^ Furuhata’s Index.

**Table 4:** Fingerprint pattern & ridge count distributions among the ABO & Rh blood types, and among the two sexes.

Group	Distribution (%)			Ridge Counts (Mean)		
	A^a^	L^b^	W^c^	TFRC	AFRC	RRC
**A**	5.7	60.3	34	120	141	24
**B**	3.8	39.5	56.7	155	224	74
**AB**	16.7	56.7	26.7	116	147	38
**O**	6.1	60.5	33.4	146	185	45
**Statistic**	χ2 (d.f^d^) = 44.236 (6)			F = 2.09	F = 3.52	F = 4.39
**p**	**< 0.0000001**			0.114	**0.022**	**0.008**
**Rh+**	5.4	55.7	38.9	136	173	41
**Rh -**	9	58	33	135	201	70
**Statistic**	χ2 (d.f) = 2.86 (2)			F = 0.002	F = 0.514	F = 2.063
**p** ^**e**^	0.239			0.962	0.477	0.157
**Males**	6.13	55.25	38.62	145	194	53
**Females**	9.7	55.33	34.97	113	139	29
**Statistic**	χ2 (d.f) = 0.986 (2)			F = 20.38	F = 19.39	F = 12.39
**p**	0.611			**<0.0001**	**<0.0001**	**<0.0001**

Fingerprint patterns differences were significant for the ABO blood types, with the AB blood type scoring the highest Arch frequencies, and the B blood type individuals scoring higher Whorl & lower Loop frequencies. Rh blood types showed little differences for both variables. Pattern differences between the sexes were minimal. Males scored higher ridge counts on all 3 variables.

^a^ Arch.

^b^ Loop.

^c^ Whorl.

^d^ Degrees of Freedom.

^e^ Significance.

The means of the ridge count variables of TFRC, ARC, & RRC scored were 129.55, 175.68, & 43.55 respectively. Further, it was shown that the TFRC of the population was normally distributed with a slight negative skew (Table 1).

### Ethnic Affiliation Related

Pattern distributions differences in the various ethnic groups, which returned highly significant differences between the groups at the 5% level (χ^2^, p<0.0000001), have revealed that the Nilo-Saharan Berta manifested significantly higher Arch frequencies (18.68%), which is high both with respect to the other studied local ethnic groups (having arch frequencies less than 7%), as well as relative to other studied populations worldwide [3, 4]. Further, the two Semitic groups (Amhara & Tigray) have been shown to manifest comparably similar frequencies for all three pattern types, indicating their similarity as populations. Similarly, the Cushitic & Omotic representatives (the Oromos & Shinashas respectively) manifested relatively similar Arch & Whorl frequencies.

Regarding the Loop Pattern, the highest frequencies are seen for the Semitics (65.82% for the Amharas, 65.96% for the Tigray), while the least frequencies were observed for the Oromo population (41.44%). Considering the Whorl pattern, the highest frequencies are registered for the Oromo population (54.33%), while the two Semitic groups share very similar frequencies, and register the second lowest frequencies (around 27.5%), next to the Nilo-Saharan (Berta) group, manifesting a Whorl frequency of 25.3%.

The mean distribution of PII in the language families has revealed that the Afro-Asiatic population has a mean PII value that is almost about 13.5, while the mean PII of the Nilo-Saharan population was found to be 10.66. Considering the groups separately, the highest PII values were recorded for the Cushitic representatives (15.01), namely the Oromos, and the lowest PII values were observed for the Nilotic representatives (10.66), namely the Berta. Such extremes in PII values, especially for that of the Cushitic Oromo group, can be hypothesized to implicate two demographic events. First of all, as postulated by Rife [4], high & low PII values (generally above 15 & below 10) implicate relatively ancient populations. Historically, the Oromo population can be traced back as to have an ancient presence in Africa [25]. Further, as can be seen from Rife’s [4] world map for mean PII values of various populations, in the African continent, PII distributions show a characteristic trend of increase on going from south to north, and thus, values above 13.5 are generally found to occur in the northern parts of the continent, further strengthening the hypothesis of the ancient historical presence of the Oromos in North Eastern Africa [25]. From another perspective, this result might also be attributable to admixture events presumed to have originated from the Levant approximately 3 KYA, involving the Oromo population, and with the effect of subsequent events such as the Islamic expansion noted to have impacted them and the North Africans likewise [9]. Given the similarity in PIIs between the Oromo group (15.01) with North Africans (13.5–14.5) [4], as well as these two groups with some representative populations from areas of the Levant (Lebanon- 14.30; Israel- 13.87; Syria- 14.55) [4], it is possible to deduce that either one or both of the above explanations could be responsible for the higher value of the PII recorded for the Oromo population.

The mean PII value of the Nilo-Saharan Berta group coincides with the expected range for areas in the African continent around North-central & central Africa and upon close observation of the linguistic distribution of certain areas in this range, it can be seen that some parts of these areas are those where Nilo-Saharan languages flourished [26]. This in turns shows that this value too is in correspondence with the general expectations. The Semitic group, on the other hand, manifested a PII of 12.10, with the Amhara & Tigray components manifesting comparable PII values of 12.08 & 12.14 respectively. Similarly, the Omotic Shinasha group manifests a PII of 13.95. These values are in concordance with the expectations one would have considering a study done on another Semitic ethnic cohort from Ethiopia [10].

Regarding the TFRC, ARC, & RRC distributions among the Ethnic groups, the ANOVA result was highly significant (p <0.0001 in all three cases). The sampled Oromo group was shown to manifest the highest values for all three ridge count variables (157.42, 235.58, and 81.61 respectively). In contrast, the sampled Berta group was shown to manifest the lowest values for the four RCs (90.04, 101.16, and 13.56 respectively). The sampled Shinasha group, as emphasized in the previous paragraphs, showed some similarities with the sampled Oromo group in manifesting comparable mean values distributions for the three ridge count variables (146.71, 188.38, 46.71 respectively). Similarly the two sampled Semitic Amhara & the Tigray groups were shown to manifest similar ridge count variable values (123.91, 149.35, & 27.70 for the Amharas, and 125.08, 146.46, & 24.21 for the Tigray group, respectively).

Post-hoc comparisons in the groups for the TFRC have revealed the Berta group manifested lower mean values as compared to the other groups (Bonferroni, p = 0.011, <0.0001, 0.007, <0.0001 when compared to the Amhara, Shinasha, Tigray, and Oromo groups respectively). Similarly, the Oromos have been shown to manifest the highest mean values, with statistically significantly differences noted between this group and all others except the Shinashas (Bonferroni, p = 0.007, 0.010 when compared to the Amharas, and the Tigray). Such comparisons on the ARC differences have revealed that the mean differences are significant for differences between the Bertas & Shinashas (Bonferroni, P<0.0001), as well as between the Oromos and three other groups: Amharas, Bertas, & Shinashas (Bonferroni, P<0.0001 in all three cases).

Finally, Post-hoc comparisons for the RRC mean differences between the ethnic groups has shown that the Berta significantly differ from the Shinasha, the latter manifesting higher means for the RRCs (Tamhane’s T2, p = 0.013). Similarly, the sampled Oromos were shown to score higher mean RRC values compared to all the other groups (Tamhane’s T2, p<0.001 when compared to the Amhara, Berta, & Tigray groups, and p = 0.022 when compared to the Shinasha group).

### Gender Related

Considering the pattern distributions, the males & females sampled in this study have been shown to manifest quite similar pattern frequencies, as seen from the statistically non-significant findings (χ^2^, p = 0.6), with only slight variations occurring for the arch & whorl pattern frequencies. The Arch, Loop, and Whorl frequencies were found to be 6.13, 55.25, & 38.62 respectively in males, and 9.7, 55.33, & 34.97 respectively in the females sampled. Further, the mean PII of males was shown to be higher (13.25), as compared to a female (10.66).

In contrast to the pattern distribution, the differences in ridge counts distributions in the two sexes were found to be statistically significant, with highly significant ANOVA results (p<0.001 for all 3 RCs). Males manifested higher RC values, with mean values for the TRC, ARC, & RRC variables being 145, 194, and 53 respectively, in contrast to the females, which scored mean values of 113, 139, 111, & 29 respectively. Such gender differences are commonly observed for Dermatoglyphic RCs across populations. Eventhough the sole genetic contributors to these quantitative traits have been shown to be predominantly autosomal [12], the effects of the sex chromosomes on RC values have also been noted, explained after observations of chromosomal aberrations such as Turner’s syndrome with a sex chromosome dosage effect on RC values [1, 12].

### ABO Blood Type Related

Pattern distributions among the ABO blood types yielded statistically significant results at the 5% level (χ^2^, p < 0.0000001). Blood types A, AB, & O share common features in that the most common & the least common patterns seen were respectively the Loop and the Arch, which is expected. In contrast, it has been observed that the Whorl pattern was more common than the Loop pattern in blood type B individuals, which contradicts to the observations made in another studied population [27]. Mean PII value differences were shown to occur among the ABO blood types, the mean PII of the B Blood type being considerably higher which contradicts (15.29) compared to the other blood types, while the lowest value was recorded for the AB blood type (11.00). The A and O blood types scored comparable mean PIIs (12.83 & 12.73 respectively).

The mean ARC & RRC differences between the ABO blood groups were shown to be significant (ANOVA, p = 0.022 & 0.008 respectively), and post-hoc comparisons revealed that both could be attributed to the differences between the A and B blood types (for ARC- Bonferroni, p = 0.019; for RRC- Tamhane’s T2, p = 0.050), with B blood type individuals scoring significantly higher ARC & RRC means.

### Rh D/d Blood Type Related

Non-significant differences were observed for the distribution of patterns among the Rh blood types (χ^2^, p = 0.239). The Rh positive blood type was shown to be associated with a slightly higher PII value (13.34) compared to the Rh negative blood type (12.40). Similarly, the TFRC, ARC, and RRC distributions among the Rh positive & negative individuals revealed only slight differences, with non-significant ANOVA results for all three variables.

## Diversity

### Allelic Frequencies

The Thumb & Finger pattern genotypes were determined and used to calculate the allelic frequencies for the 7 polymorphic loci (Table B in S1 File), employed to further analyze diversity & infer group similarities.

### Heterozygosity (Gene Diversity), Fixation. & Panmixia

The Heterozygosities for the five groups (Table 5) varied from 0.1978 to 0.1250. It is established that the genetic heterozygosity of a population is favoured by out-breeding, while homozygosity is increased by inbreeding within a population during the course of evolution. Further, higher fitness has also been shown for groups with higher heterozygous genetic make-ups, as compared to homozygous inbreeding endogamous populations, generally increasing the chance of manifestation of deleterious alleles. In line with this, F estimates compare the extent of inbreeding for populations during evolution, while P values signify the extent of out-breeding for a locus in a population.

**Table 5:** Heterozygosity (H), Fixation Index(F), & Panmictic Index (P) for the Ethnic Groups computed from 2 polymorphic Loci.

Group	H	F	P
**Amhara**	0.1978	0.5856	0.4144
**Berta**	0.1765	0.6412	0.3588
**Oromo**	0.1250	0.6785	0.3215
**Shinasha**	0.1452	0.6970	0.3030
**Tigray**	0.1754	0.6241	0.3759
**Mean**	**0.1640**	**0.6453**	**0.3547**

High heterozygosity & panmictic index has been noted for the Amhara ethnic group, indicating higher diversity. Lower values for heterozygosity in the other groups in turn indicate more homogeneity of these groups.

In the current study, the highest value for heterozygosity, which was recorded in the Amhara population, indicates that this population has a relatively higher genetic variation (is more diversified). Further, the Oromo population has recorded the least value for heterozygosity, followed by the Shinasha group. Also, the Oromo population simultaneously manifested one of the highest fixation index (F) values (lowest panmixia, P), next to the Shinashas, suggesting more homozygosity and thus, higher homogeneity in this group relative to the other studied groups.

### Nei’s measure of genetic diversity (GST)

The gene diversity (Table 6) among the five ethnic groups (H_T_ = 0.1760) has been analyzed into its two component: Intra-population gene diversity (H_S_ = 0.1637), and Inter-population gene diversity (D_ST_ = 0.0123), which shows a very low inter population gene diversity as compared to the intra population gene diversity. Researchers analyzing Loci related to blood groups for some human populations [28] observed such trends and attributed them to the fact that in such instances, only a small fraction of the total population gene diversity occurs due to the differences between the population groups, while a large fraction of this diversity occurs due to individual variations, i.e., within the population groups. Further the value of G_ST_ = 0.0450 shows that only about 4.5% of differentiation among populations exists, which is relatively very low compared to other populations [29].

**Table 6:** Estimates of Nei’s measures of gene diversity among the ethnic groups based on 7 polymorphic Loci.

Locus	D_ST_	H_T_	H_S_	G_ST_
**A**	0.0434	0.4983	0.4550	0.0870
**B**	0.0007	0.0493	0.0487	0.0133
**C**	0.0005	0.0445	0.0440	0.0121
**C(2)**	0.0308	0.4951	0.4643	0.0623
**D**	0.0099	0.1082	0.0983	0.0915
**E**	0.0002	0.0189	0.0186	0.0127
**F**	0.0007	0.0179	0.0172	0.0364
**Mean**	**0.0123**	**0.1760**	**0.1637**	**0.0450**

A low inter population gene diversity relative to the intra population gene diversity can be seen, implying that only a small fraction of the total population gene diversity occurs due to the differences between the population groups, while a large fraction of this diversity occurs due to individual variations, i.e., within the population groups.

### Wahlund’s variance

Both the individual values, and the mean values for Wahlund’s variance (f) are generally high (Table 7), relative to findings of researchers analyzing loci related to blood groups for some ethnically divided human populations [28,29].

**Table 7:** Mean allelic frequencies with their variance & Wahlund’s *f* estimates for the Ethnic Groups computed from seven polymorphic fingerprint pattern related Loci.

Allele (Locus)	Mean (P)	Variance (σP^2^)	Wahlund’s Variance (*f*)
**A**	0.4711	0.0217	0.0870
**B**	0.0253	0.0003	0.0133
**C**	0.0228	0.0003	0.0121
**C (2)**	0.5493	0.0154	0.0623
**D**	0.9426	0.0049	0.0915
**E**	0.0095	0.0001	0.0127
**F**	0.0090	0.0003	0.0364
**Mean**	**0.2899**	**0.0062**	**0.0450**

The estimations have revealed that both the individual values, as well as the mean values for Wahlund’s variance (f) are generally high for the sampled population.

### Genetic Distance & Phylogeny

**Fig 2:**
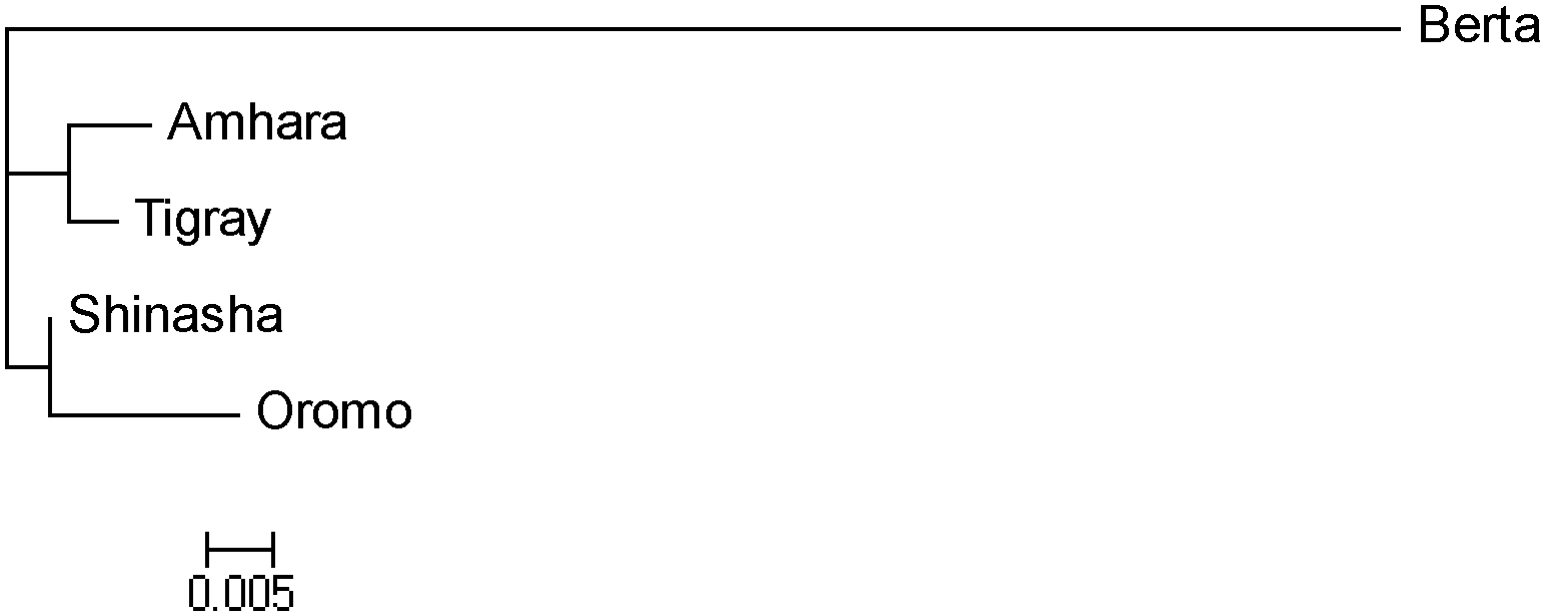
Neighbour Joining Tree for the studied groups. The relative similarity between the studied groups inferred from the inheritance model of Slatis [16] & pattern frequencies. The two Semitic groups (Amhara, Tigray) have clustered out together, as have the Cushitic (Oromo) group that fall under a broader cluster of the Afro-Asiatics. The Nilo-Saharan Berta group, being from a different linguistic family, has clustered out separately.

Based on the proposed inheritance model of Slatis [16], and following recent studies that have tried to apply it for the estimation of population diversity and differentiation, as well as homology inferences for subdivided populations [30], the gene frequencies based on the Dermatoglyphic phenotypes were calculated, from which Nei’s D_A_ distance matrix was estimated (Table 8), and a neighbour joining (NJ) tree was constructed (Fig 2). This has ascertained the so far similarities observed between the two Semitic populations (Amhara, Tigray) which clustered out together, with the Cushitic Oromo population falling within a broader Afro-Asiatic cluster. Likewise, similar results were alos observed for the Omotic Shinasha population. In addition, the NJ tree inferred for this study was found to be in concordance to that of whole genome sequencing & related analyses of Ethiopian populations [9].

**Table 8:** Distance Matrix generated as Nei’s standard D_A_ distance from 7 polymorphic loci.

	Berta	Oromo	Shinasha	Tigray
**Amhara**	0.120	0.020	0.017	0.010
**Berta**		0.124	0.105	0.110
**Oromo**			0.013	0.031
**Shinasha**				0.008

The two Semitic groups (Amhara & Tigray) have been shown to be less distant (0.010), while the Nilo-Saharan Berta and the Cushitic Berta were found to be the most distant. Distances between the Cushitic & semitic, as well as the Omotic groups, all under the Afro-Asiatic Cluster, were relatively insignificant.

## Conclusions

The study shows the application of Dermatoglyphics research in various aspects of population genetics & anthropology, in concordance to recommendations from previous works [5–7]. It additionally evaluated prospects of developing a method to trace back population origins & ancient movement patterns to a certain degrees by coinciding historical accounts with Dermatoglyphics parameters. Further, the study perfectly agrees with the genetic studies done for this population [9], which have deemed that linguistic clustering is a significant factor for the Ethiopian population. In contrast, other global population findings from genetic & Dermatoglyphic studies have instead revealed geographic patterning as to being more important to intra-population genetic differentiation, as in the case of the Chinese population [7, 31]. This in turn entails several important implications, the first of which is that it necessitates in having an initial knowledge as to the type of stratification existing within groups for a given population before conducting any further genetic diversity & differentiation studies. Secondly, it highlights on a potential of using Dermatoglyphic markers as relatively simple, non-invasive, and inexpensive preliminary tools applicable prior to conducting complex intra-group genetic diversity & differentiation studies on populations. Finally, the researchers suggest to those aiming to pursue further genetic studies in Ethiopian populations to take into consideration the importance of linguistic clustering, thus assigning larger groupings based on common linguistic affiliations of population sub-groups.

## Supporting Information

S1 File **Table A,** Inheritance model for the fingerprint pattern. 7 genes related to fingerprint patterns. **Table B,** Allelic Frequencies. Allelic frequencies among the ethnic groups.
(DOCX)
