# Supplementary material for: Ethiopian Population Dermatoglyphic Study Reveals Linguistic Stratification of Diversity
Source: PLoS One. 2015 Jun 4;10(6):e0126897. doi: 10.1371/journal.pone.0126897 (PMC4456081; doi:10.1371/journal.pone.0126897)
Supplement: S1 File — Table A, Inheritance model for the fingerprint pattern. 7 genes related to fingerprint patterns. Table B, Allelic Frequencies. Allelic frequencies among the ethnic groups. (DOCX) [file pone.0126897.s001.docx]

# Table A- Inheritance model for the fingerprint pattern

| **Locus** | **Gene** | **Genotype** | **Phenotype** |
| --- | --- | --- | --- |
| 1 | Semi-Dom. | AA | Whorls on both thumbs |
|  |  | Aa | 2 ulnar loops, or 1 ulnar loop & one whorl |
|  |  | aa | Ulnar loops on both thumbs |
| 2 | Dominant | C- | Radial loops on index, often with arches on middle fingers |
| 3 | Dominant | B- | Arches on both thumbs, & often on other (but not all) fingers |
| 4 | Semi-Dom. | CC | Whorls on both ring fingers |
|  |  | Cc | Two ulnar loops or one ulnar loop & one whorl |
|  |  | cc | Ulnar loop on both ring fingers |
| 5 | Recessive | dd | Radial loops on ring or little finger |
| 6 | Dominant | E | Whorls on all except for an ulnar loop on the middle finger |
| 7 | Dominant | F- | Arches on all fingers |

7 genes related to fingerprint patterns

# Table B- Allelic frequencies

|  |  | **Allele Frequencies** | | | | |
| --- | --- | --- | --- | --- | --- | --- |
| **Loci** | **Alleles** | **Amhara** | **Berta** | **Oromo** | **Shinasha** | **Tigray** |
| 1 | **A** | 0.3412 | 0.4333 | 0.7050 | 0.5667 | 0.3091 |
|  | **a** | 0.6588 | 0.5667 | 0.2950 | 0.4333 | 0.6909 |
| 2 | **C** | 0.4607 | 0.4386 | 0.7598 | 0.6230 | 0.4643 |
|  | **c** | 0.5393 | 0.5614 | 0.2402 | 0.3770 | 0.5357 |
| 3 | **B** | 0.0222 | 0.0607 | 0.0097 | 0.0163 | 0.0177 |
|  | **b** | 0.9778 | 0.9393 | 0.9903 | 0.9837 | 0.9823 |
| 4 | **C** | 0.0166 | 0.0529 | 0.0097 | 0.0081 | 0.0276 |
|  | **c** | 0.9834 | 0.9471 | 0.9903 | 0.9919 | 0.9733 |
| 5 | **D** | 0.8512 | 1.0000 | 0.8613 | 1.0000 | 1.0000 |
|  | **d** | 0.1482 | 0.0000 | 0.1387 | 0.0000 | 0.0000 |
| 6 | **E** | 0.0000 | 0.9701 | 0.0097 | 0.0081 | 0.0000 |
|  | **e** | 1.0000 | 0.0299 | 0.9903 | 0.9919 | 1.0000 |
| 7 | **F** | 0.0000 | 0.0451 | 0.0000 | 0.0000 | 0.0000 |
|  | **f** | 1.0000 | 0.9549 | 1.0000 | 1.0000 | 1.0000 |

Allelic frequencies among the ethnic groups
